# The bidirectional modulation of acute aerobic exercise on cognitive performance and brain electrical activity in adolescents with smartphone addiction: an experimental study using EEG power spectrum analysis

**DOI:** 10.3389/fpubh.2026.1720007

**Published:** 2026-02-25

**Authors:** Tongtong Che, Yanni Long, Shuangshuang Zhang, Xianglong Jiang

**Affiliations:** 1 School of Physical Education, Qingdao University, Qingdao, China; 2 Qingdao Preschool Education College, Qingdao, China

**Keywords:** acute aerobic exercise, adolescents, brain electrical activity, cognitive control, cognitive function, EEG, smartphone addiction

## Abstract

**Objective:**

Smartphone addiction has become a significant mental health issue among adolescents, affecting their cognitive functions and social adaptability. Although acute aerobic exercise has been shown to improve cognitive performance, its effects on adolescents with smartphone addiction remain unclear. This study aims to explore the impact of acute aerobic exercise on cognitive performance and brain electrical activity in adolescents with smartphone addiction and to elucidate its underlying neural mechanisms.

**Methods:**

A randomized controlled experimental design was adopted, with 40 male adolescents with smartphone addiction recruited and randomly assigned to either the experimental group (acute aerobic exercise) or the control group (neutral reading condition). Cognitive performance was assessed using the Stroop task and the 2-back task, while EEG was used to record brain electrical activity, focusing on changes in the power of α, β, and θ waves.

**Results:**

The experimental group showed significant improvements in accuracy (*p* < 0.001, *d* = −2.67) and reaction time (*p* < 0.001, *d* = 3.17) in the Stroop task, and in accuracy (*p* < 0.001, *d* = −3.26) and reaction time (*p* < 0.001, *d* = 3.29) in the 2-back task. EEG results revealed that acute aerobic exercise significantly increased the power of α and β waves in the prefrontal, central, and parietal regions (*p* < 0.001), while θ wave power in the prefrontal and central regions significantly decreased (*p* < 0.05).

**Conclusion:**

Acute aerobic exercise significantly enhanced the cognitive performance of adolescents with smartphone addiction, particularly in attention and working memory tasks. EEG analysis indicated that exercise optimized the cognitive control network by enhancing α and β wave power and suppressing θ wave power. These findings provide empirical support for exercise interventions to improve cognitive deficits in adolescents with smartphone addiction.

## 1 Introduction

With the rapid development of digital information technology and the widespread use of smartphones, smartphone addiction has become an increasingly prominent psychological health issue among adolescents. Its rising prevalence and negative consequences have garnered growing attention (1). Smartphone addiction is typically characterized by compulsive overuse of smartphones, accompanied by tolerance, withdrawal symptoms, and significant impairment in real-life functioning (2, 3). An increasing body of research has shown that smartphone addiction not only leads to distractibility but also causes declines in executive functions and working memory (4). Deficits in these core cognitive functions can have lasting and profound negative effects on adolescents' academic performance, social adaptation, and mental health. Therefore, there is an urgent need to explore effective intervention strategies.

In recent years, non-pharmacological interventions have gained widespread attention from psychologists and neuroscientists. Among these, aerobic exercise has been recognized as a safe and easy-to-implement intervention with significant effects on improving cognitive function (5). A substantial body of empirical evidence suggests that even brief bouts of acute aerobic exercise can significantly improve attention levels and working memory performance (6, 7), as well as promote overall executive function enhancement. Recent evidence has further identified physical activity as a critical protective factor that can directly decrease the tendency toward smartphone addiction, highlighting the necessity of integrating exercise into prevention protocols (8). However, whether these positive effects extend to adolescents with smartphone addiction remains underexplored, with a lack of systematic and in-depth studies.

From the perspective of cognitive neuroscience, both attention control and working memory processing are closely linked to prefrontal cortex (PFC) function (9). The PFC plays a critical role in high-level cognitive processes such as cognitive control, inhibition, and the maintenance and updating of working memory. Dysfunction in the PFC can significantly affect an individual's learning and social interactions (10). Existing evidence suggests that aerobic exercise can effectively activate prefrontal neural activity, enhance blood flow, and improve neuronal function, thereby facilitating attention control and working memory performance (11, 12). Moreover, randomized controlled trials have demonstrated that acute moderate-intensity aerobic exercise is a feasible and reliable means to ameliorate cravings in individuals with mobile phone dependency by potentially repairing impaired inhibitory control (13). However, it remains unclear whether these improvements can be replicated in adolescents with smartphone addiction, and whether the underlying neural mechanisms align with those observed in the general population.

Therefore, this study employs a randomized controlled experimental design, combining behavioral paradigms (2-back and Stroop tasks) with EEG frequency band power analysis, to comprehensively examine the effects of acute aerobic exercise on attention and working memory in adolescents with smartphone addiction. Based on previous literature and the research objectives, we propose the following research hypotheses: (1) acute aerobic exercise will significantly enhance cognitive performance in smartphone-addicted adolescents, evidenced by increased accuracy and reduced reaction times in both the Stroop and 2-back tasks; (2) acute aerobic exercise will induce a bidirectional modulation of brain electrical activity, specifically characterized by an increase in α and β wave power and a decrease in θ wave power in the prefrontal and central regions. This research aims to only help clarify the scientific rationale for using exercise to address cognitive deficits associated with smartphone addiction, but also provide valuable theoretical support for developing targeted interventions to enhance adolescent mental health.

## 2 Participants and methods

### 2.1 Participants

Volunteers were recruited through the Qingdao University online platform and campus announcements, with an initial total of 63 male students voluntarily enrolling in the study. These participants completed the Smartphone Addiction Scale-Short Version (SAS-C) and underwent a thorough physical health screening to ensure they met the experimental criteria and could safely participate in the aerobic exercise intervention. Based on the inclusion and exclusion criteria, 40 male participants who met the conditions were selected for the study. Participants were randomly assigned to either the experimental group or the control group using a random number table, with 20 participants in each group. The sample size was determined based on an *a-priori* power analysis using G^*^Power 3.1.9.7. For a 2 × 2 mixed-design ANOVA (group × time), assuming a medium effect size (*f* = 0.25), an α level of 0.05, and a power (1–β) of 0.80, the minimum required total sample size was calculated to be *N* = 34. To ensure strictly balanced group sizes and to further enhance the robustness and reliability of the statistical findings, we finalized the study with 40 participants (20 per group), providing sufficient and trustworthy statistical power to detect significant effects.

The inclusion criteria were: 1) male, aged 18 to 25 years, right-handed; 2) SAS-C score higher than 77 points; 3) physically healthy, with no significant cardiovascular, respiratory, or neurological disorders, and no contraindications to exercise; 4) normal vision (including corrected vision) and able to perform the visual identification tasks required by the experiment; 5) voluntary participation with signed informed consent. The exclusion criteria were: 1) individuals with mental disorders or currently receiving psychiatric treatment; 2) individuals taking medications that may affect cognitive function or mood; 3) individuals with severe sleep disorders, alcohol abuse, or a history of substance abuse; 4) individuals who had participated in other exercise or psychological intervention studies within the past 3 months.

The study protocol was approved by the Ethics Committee of Qingdao University College of Medicine (Ethical approval number: QDU-HEC-2025356). The entire experimental procedure adhered strictly to the ethical principles outlined in the Declaration of Helsinki. Prior to participation, all participants were fully informed about the study's purpose, content, and potential risks, and all participants voluntarily provided written informed consent.

### 2.2 Experimental procedure

This study utilized a Randomized Controlled Trial (RCT) design. The overall procedure consisted of a baseline testing phase (T1) and a post-intervention testing phase (T2), with a one-week interval between them to minimize fatigue and practice effects. All experiments began at 10:00 a.m.,s and were conducted in the Psychology Laboratory at Qingdao University. The laboratory environment was quiet, with ambient lighting, and the temperature was maintained at 22–25 °C with 45%−55% humidity, ensuring minimal external distractions.

During the baseline testing phase (T1), participants first rested quietly for 5 min, while resting EEG, heart rate, and blood pressure were recorded. They then completed the Stroop task and the 2-back task, with the task order counterbalanced between participants to avoid order effects. During the experiment, EEG data and behavioral indicators (reaction time and accuracy) were collected concurrently.

In the intervention and post-intervention testing phase (T2, one week later), the experimental group performed 20 min of acute moderate-intensity aerobic exercise, while the control group engaged in 20 min of neutral reading. The exercise intensity was kept within 60%−75% of each participant's maximum heart rate, calculated as (220–age), and was monitored using heart rate and the Rating of Perceived Exertion (RPE) scale (12–14) to ensure that the intensity remained moderate. After the intervention, both groups rested quietly for 10 min to allow their physiological measures to return to baseline levels. Following this, participants rested again for 5 min, during which EEG, heart rate, and blood pressure were recorded. They then completed version B of the tasks, which were difficulty-matched but with different materials (e.g., Stroop using different color words and 2-back using different letter sequences). The task order was again counterbalanced across participants. Throughout the experiment, EEG signals and behavioral data were collected simultaneously. At the conclusion of the experiment, participants completed the Positive and Negative Affect Schedule (PANAS) to assess changes in emotional state following the acute aerobic exercise.

**Figure 1:**
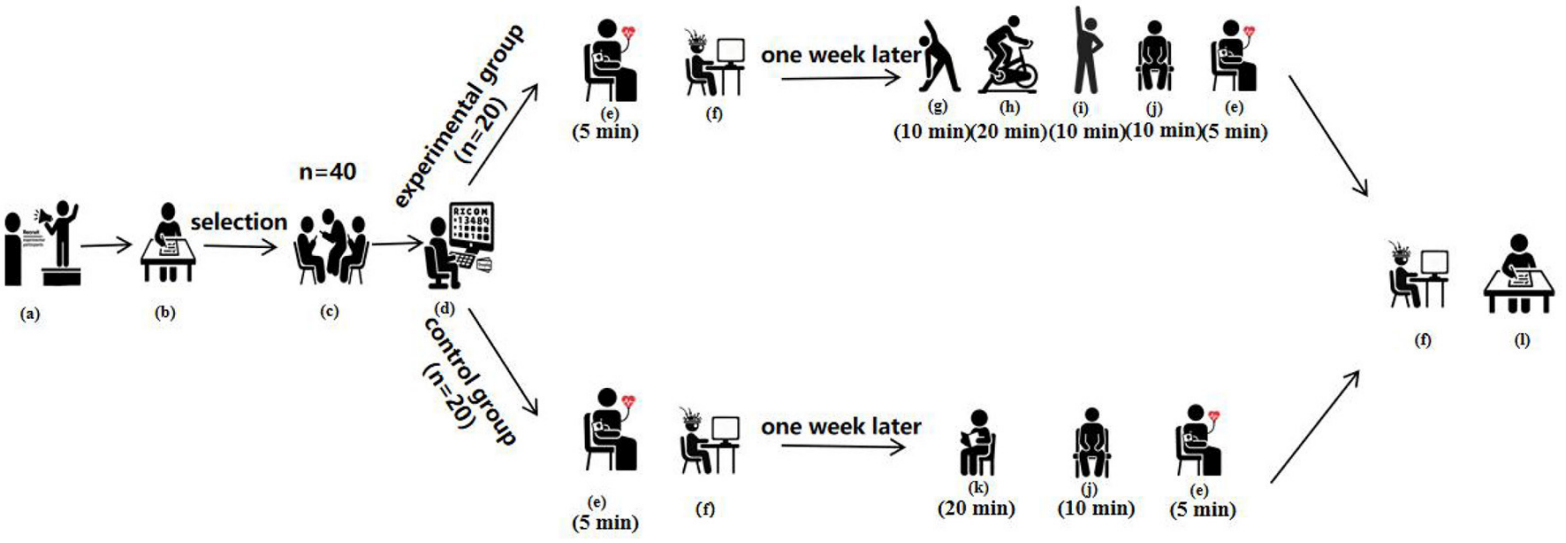
Experimental flowchart. a: Volunteer recruitment; b: Completion of the Smartphone Addiction Scale (SAS-C); c: Participants; d: Random assignment;e: Resting heart rate and blood pressure measurement; f: Stroop and 2-back tasks; g: Warm-up; h: Moderate-intensity aerobic exercise intervention; i: Stretching; j: Resting; k: Neutral reading task; l: Completion of the positive and negative affect schedule (PANAS).

The entire experimental procedure was strictly followed by trained researchers to ensure scientific validity and data integrity, as illustrated in Figure 1.

### 2.3 Test content

To examine the immediate effects of acute aerobic exercise on attention, working memory, and emotional state in adolescents with smartphone addiction, this study integrates psychological experimental paradigms with electroencephalogram (EEG) measurements for a comprehensive assessment.

#### 2.3.1 Psychological paradigms

The tasks were presented on a Huawei MateBook 14 laptop (14-inch screen, 1,920 × 1,080 resolution, Windows 11) with an external Logitech keyboard (Switzerland) and were run using PsychoPy software.

**Figure 2:**
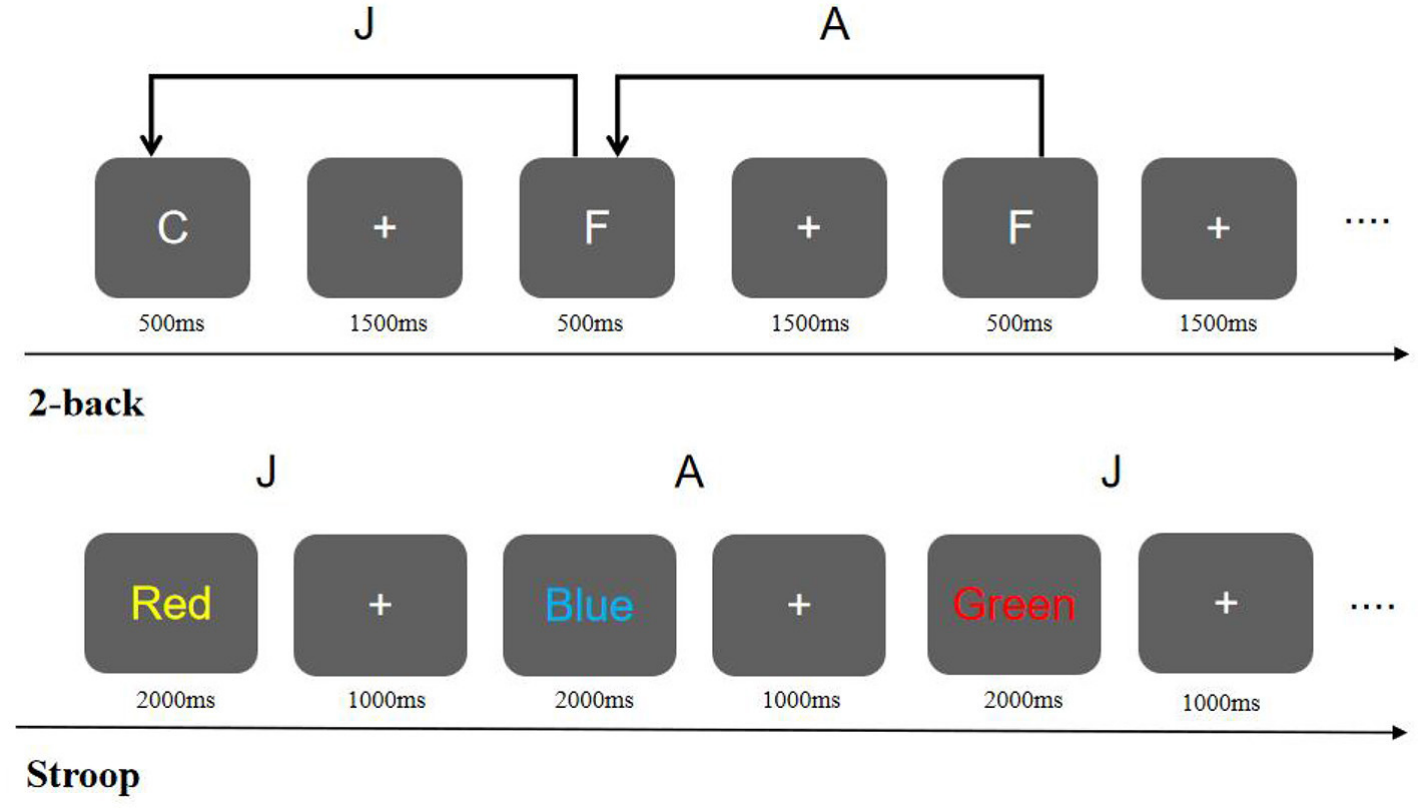
Schematic of the Stroop task and 2-back task experimental procedures.

(1) Stroop Task: The Stroop task was used to assess attention and inhibitory control. The experimental stimuli were Chinese characters “红” (red), “蓝” (blue), “绿” (green), and “黄” (yellow), presented in one of four font colors: red, blue, green, or yellow. The task consisted of congruent conditions (where the font color matched the word's meaning) and incongruent conditions (where the font color did not match the word's meaning). Each stimulus was presented for 2,000 ms, followed by a fixation point “+” for 1,000 ms as an interval. Participants were required to respond quickly by pressing a key based on the font color (e.g., press “A” for red, “J” for blue). Reaction times and accuracy were recorded. All stimuli were presented in random order, and each condition was repeated 40 times to ensure the reliability and validity of the results.

(2) 2-back Task (14): The 2-back task was used to measure working memory capacity. In the experiment, a random sequence of letters (A–J) was presented, with each letter shown for 500 ms, followed by a fixation point “+” for 1,500 ms as a response window. Participants were required to judge whether the current letter matched the one two letters prior. If they matched, participants pressed the “A” key; if they did not match, they pressed the “J” key. The target stimulus percentage was approximately 35%, and the sequence length was 60. Reaction times and accuracy were recorded. The task flow is depicted in Figure 2.

#### 2.3.2 EEG measurement and processing

**Figure 3:**
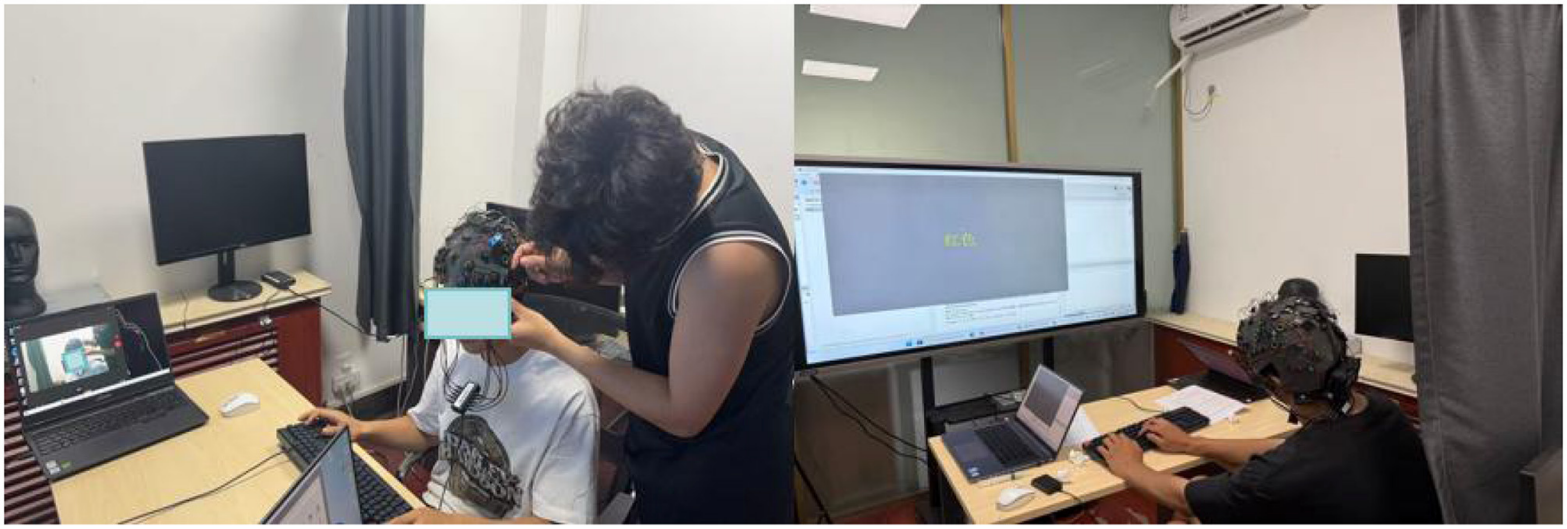
EEG experimental data collection site.

EEG data were collected using the g.Nautilus Research 32-channel wireless EEG system (g.tec medical engineering GmbH, Austria) with the g.SCARABEO and g.LADYbird active wet electrodes (Figure 3). The electrode configuration followed the international 10–20 system with a total of 32 channels. The reference electrode was placed on both earlobes, and the ground electrode was positioned near AFz. During the experiment, the impedance of all electrodes was maintained below 5 kΩ to ensure stable signal quality. EEG signals were wirelessly transmitted to the receiver via a 2.4 GHz system and monitored and stored in real-time using the g. Recorder software. The sampling rate was set at 500 Hz, with a hardware bandpass filter range of 0.01–100 Hz and a 50 Hz notch filter to remove power line noise.

Although the device supports 32-channel data collection, we selected 16 channels for analysis in this study to simplify data processing and focus on key brain areas. The primary goal of the experiment was to investigate the effects of acute aerobic exercise on the electrical activity of the prefrontal cortex (PFC) and central region, which are involved in attention control and working memory. We selected these areas because they are central to cognitive control and working memory. The 16 channels provided sufficient coverage of these important regions, allowing for efficient analysis while avoiding unnecessary redundancy from 32 channels.

Data processing was conducted in MATLAB R2022a, using the EEGLAB toolbox and custom scripts. The raw data were first bandpass filtered between 1–40 Hz to eliminate low-frequency drift and high-frequency artifacts. Independent component analysis (ICA) was applied to remove eye movement and blink artifacts. The data were then segmented according to experimental phases, extracting segments for resting state (5 min), Stroop task, and 2-back task. Fast Fourier Transform (FFT) was applied to calculate the power spectral density, and θ (4–8 Hz), α (8–13 Hz), and β (13–30 Hz) band powers were extracted. All power values were normalized as relative power percentages, and the changes between pre-test (T1) and post-test (T2) were calculated. Finally, at the channel level, the power for each of the 16 channels was computed, and average power for regions of interest (ROIs), such as the prefrontal cortex (Fp1/Fp2/Fz), central region (C3/C4/Cz), and parietal region (P3/P4), was extracted for further statistical analysis.

### 2.4 Statistical analysis

All data were analyzed using SPSS 27.0 and Excel 2021, with results presented as mean ± standard deviation (Mean ± SD). All significance tests were two-tailed, with the significance level set at 0.05.

EEG data were first extracted for the relative power of three frequency bands: θ (4–8 Hz), α (8–13 Hz), and β (13–30 Hz) in three regions of interest (ROIs): the prefrontal cortex (Fp1, Fp2, Fz), central region (C3, C4, Cz), and parietal region (P3, P4). The average power across channels within each ROI was used as the indicator for that region.

Behavioral data included the average reaction time (RT), accuracy, and Stroop interference effect for the Stroop task, as well as accuracy and average reaction time for the 2-back task. These data were analyzed using a 2 (group: experimental group vs. control group, between-subjects factor) × 2 (time: T1 vs. T2, within-subjects factor) mixed repeated-measures ANOVA to test the main effects of time, group, and their interaction. For significant effects, simple effects analyses were performed using paired sample *t*-tests and independent sample *t*-tests.

Additionally, to visualize the EEG changes before and after the intervention, percentage change in EEG data (Δ % = (T2 – T1)/T1 × 100%) was calculated, and topographic maps were plotted. The Δ % results were used for visualization purposes and were not considered as the primary statistical indicators.

For effect sizes, partial η^2^ (η^2^) was reported for the ANOVA results, where 0.01, 0.06, and 0.14 represent small, medium, and large effects, respectively. Cohen's *d* was reported for *t*-test results, where 0.20, 0.50, and 0.80 represent small, medium, and large effects, respectively. *Post hoc* comparisons were further analyzed using Bonferroni correction. For multiple comparisons across similar indicators, False Discovery Rate (FDR) control was applied to reduce the Type I error rate.

## 3 Results

### 3.1 Changes in Stroop and 2-back Task Performance

Repeated measures ANOVA revealed significant time main effects for Stroop accuracy (*p* < 0.001), Stroop reaction time (*p* < 0.001), 2-back accuracy (*p* < 0.001), and 2-back reaction time (*p* < 0.001), indicating that acute aerobic exercise significantly improved performance on these tasks. The Stroop interference effect accuracy (*p* > 0.05) showed no significant difference, while reaction time did show a significant difference (*p* < 0.05). No significant differences were observed for the group main effects (*p* > 0.05), indicating no significant differences in task performance between the experimental and control groups. The group × time interaction was significant for Stroop accuracy (*p* < 0.001), Stroop reaction time (*p* < 0.001), 2-back accuracy (*p* < 0.001), and 2-back reaction time (*p* < 0.001), with the experimental group showing more pronounced effects of acute aerobic exercise.

Paired sample *t*-test results showed significant improvements in the experimental group for Stroop accuracy (*p* < 0.001, Cohen's *d* = −2.667, very large effect), Stroop reaction time (*p* < 0.001, Cohen's *d* = 3.172, very large effect), 2-back accuracy (*p* < 0.001, Cohen's *d* = −3.259, very large effect), and 2-back reaction time (*p* < 0.001, Cohen's *d* = 3.292, very large effect). In contrast, the control group showed no significant changes across any of the measures, with effect sizes ranging from small to medium (Stroop accuracy: Cohen's *d* = 0.161, small effect; Stroop reaction time: Cohen's *d* = −0.172, small effect; 2-back accuracy: Cohen's *d* = 0.091, small effect; 2-back reaction time: Cohen's *d* = 0.135, small effect), indicating minimal changes in the control group.

**Figure 4:**
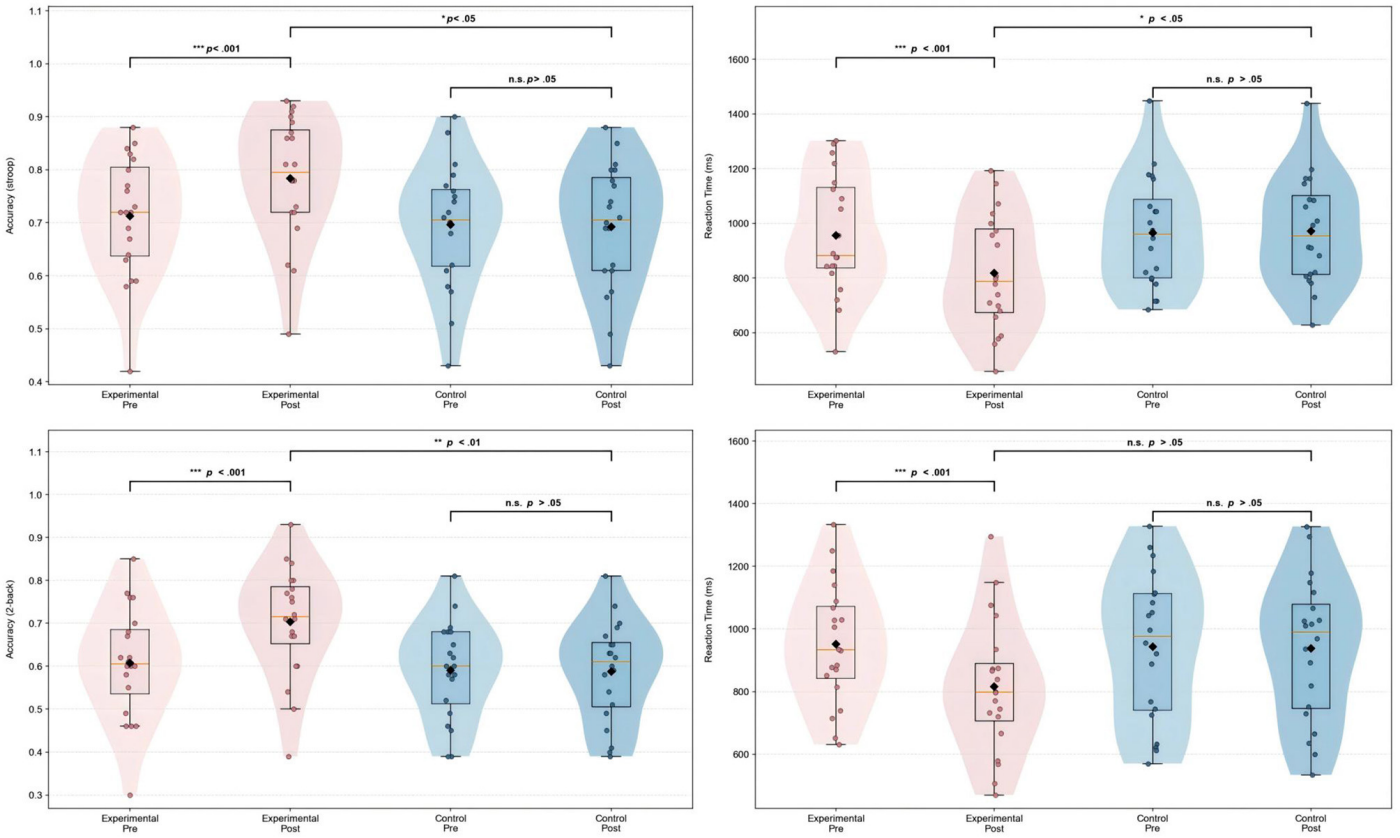
Changes in Stroop and 2-back task performance (accuracy and reaction time) between experimental and control groups.

Independent sample *t*-test results showed significant differences between the experimental and control groups in post-test Stroop accuracy (*p* < 0.05, Cohen's *d* = 0.772, medium effect), Stroop reaction time (*p* < 0.05, Cohen's *d* = −0.756, medium effect), and 2-back accuracy (*p* < 0.01, Cohen's *d* = 0.934, large effect), while no significant difference was found for 2-back reaction time (*p* > 0.05, Cohen's *d* = −0.551, medium effect). The experimental group performed better on multiple measures of accuracy and reaction time, particularly in 2-back accuracy, where the experimental group outperformed the control group significantly, as shown in Table 1 and Figure 4.

**Table 1:** Means, standard deviations, and ANOVA results for the Stroop and 2-back task performances in the experimental and control groups.

**Measure**	**Group**	**Pretest (M ±SD)**	**Posttest (M ±SD)**	Main Effect (partial ***η***^**2**^)		***F* [group] × [time] (partial η^2^)**
				**Group**	**Time**	
Stroop accuracy	Experimental	0.713 ± 0.115	0.784 ± 0.11	1.767(0.085)	78.078^***^ (0.804)	76.017^***^ (0.800)
	Control	0.696 ± 0.116	0.692 ± 0.121			
Stroop reaction time (ms)	Experimental	956.250 ± 218.890	817.300 ± 209.410	1.995 (0.095)	165.266^***^ (0.897)	104.389^***^ (0.846)
	Control	965.450 ± 203.000	971.300 ± 197.950			
Stroop interference Accuracy	Experimental	0.040 ± 0.008	0.039 ± 0.008	1.950 (0.093)	0.026 (0.001)	0.299 (0.016)
	Control	0.042 ± 0.006	0.042 ± 0.008			
Stroop interference reaction time	Experimental	64.300 ± 7.550	59.400 ± 7.780	00.006 (0.000)	5.684^*^ (0.230)	00.214 (0.011)
	Control	63.600 ± 7.760	59.850 ± 8.520			
2-back accuracy	Experimental	0.607 ± 0.131	0.704 ± 0.129	2.961 (0.135)	131.048^***^ (0.873)	143.709^***^ (0.883)
	Control	0.591 ± 0.113	0.589 ± 0.117			
2-back reaction time (MS)	Experimental	951.350 ± 193.510	815.150 ± 213.060	0.924 (0.046)	189.545^***^(0.909)	77.071^***^(0.802)
	Control	942.450 ± 236.600	937.100 ± 228.980			

^*^p < 0.05, ^**^p < 0.01, ^***^p < 0.001.

### 3.2 Changes in EEG power across different brain regions before and after intervention

Repeated measures ANOVA showed highly significant main effects of time (*p*s < 0.001) for all EEG power indicators, indicating a clear improvement in performance before and after the intervention. Specifically, α wave power (parietal, central, and, prefrontal regions), β wave power (parietal, central, and, prefrontal regions), and θ wave power (parietal, central, and, prefrontal regions) all showed significant time effects, indicating that the experimental intervention significantly improved power in these brain wave regions. For the main group effect, α wave power (parietal and central regions) showed significant differences (*p*s < 0.05), indicating that the experimental group had significantly higher power than the control group in these regions. No significant group differences were found for α wave power (prefrontal region), β wave power, and θ wave power across all regions (*p*s > 0.05), indicating no significant group differences in these areas. Significant group × time interactions were observed (*p*s < 0.001), indicating that the experimental and control groups showed different magnitudes of improvement before and after the intervention, especially in the parietal α wave power and central β wave power, where the experimental group showed significantly greater improvements.

Both the experimental and control groups showed significant pre-post differences across all EEG power indicators. α wave power (parietal region): the control group (*p* < 0.001, *d* = −2.945, very large effect) and experimental group (*p* < 0.001, *d* = −4.907, very large effect) both showed significant increases. α wave power (central region): The control group (*p* < 0.001, *d* = −3.261, very large effect) and experimental group (*p* < 0.001, *d* = −7.580, very large effect) both showed significant increases. α wave power (prefrontal region): The control group (*p* < 0.001, *d* = −2.776, large effect) and experimental group (*p* < 0.001, *d* = −8.249, very large effect) both showed significant increases. β wave power (parietal region): the control group (*p* < 0.001, *d* = −1.303, large effect) and experimental group (*p* < 0.001, *d* = −4.400, very large effect) both showed significant increases. β wave power (central region): the control group (*p* < 0.001, *d* = −1.140, large effect) and experimental group (*p* < 0.001, *d* = −5.765, very large effect) both showed significant increases. β wave power (prefrontal region): the control group (*p* < 0.001, *d* = −1.046, large effect) and experimental group (*p* < 0.001, *d* = −7.109, very large effect) both showed significant increases. θ wave power (parietal region): the control group (*p* > 0.05, *d* = −0.231, small effect) showed no significant difference, while the experimental group (*p* < 0.001, *d* = 2.381, very large effect) showed significant improvement. θ wave power (central region): the control group (*p* > 0.05, *d* = 0.357, small effect) showed no significant difference, while the experimental group (*p* < 0.001, *d* = 3.636, very large effect) showed significant improvement. θ wave power (prefrontal region): the control group (*p* > 0.05, *d* = 0.080, small to no effect) showed no significant difference, while the experimental group (*p* < 0.001, *d* = 4.552, very large effect) showed significant improvement.

**Figure 5:**
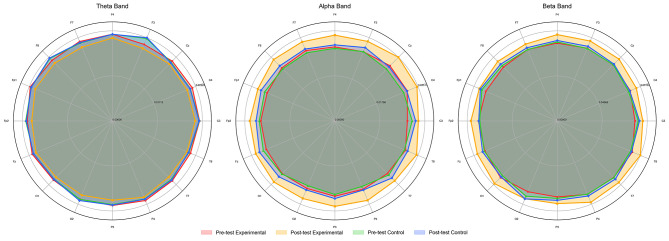
Inter-group circular distribution comparison of EEG power across different bands.

**Figure 6:**
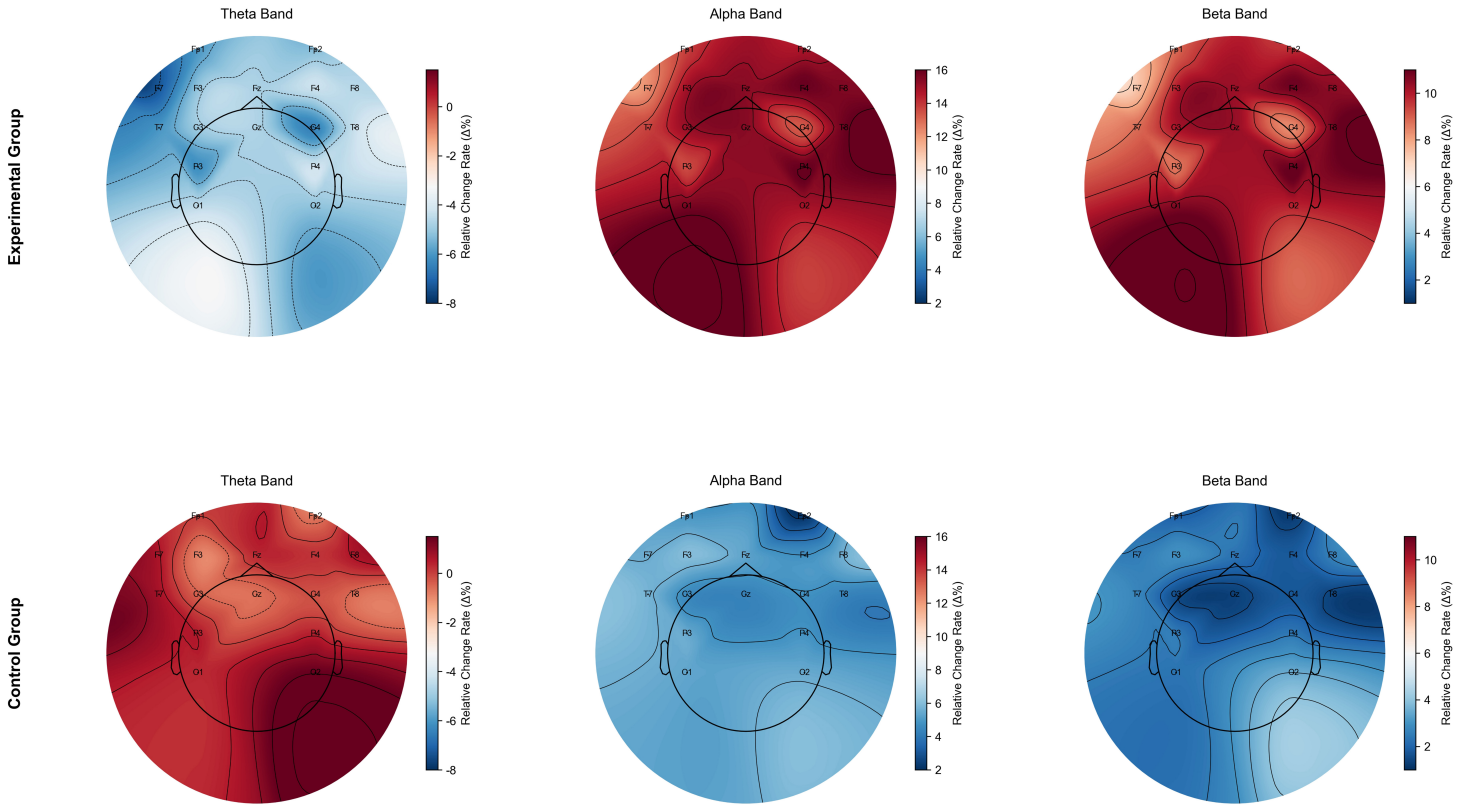
Topographical distribution of relative EEG power changes across bands in experimental and control groups.

Independent sample *t*-test results showed significant differences between the experimental and control groups for all EEG power indicators in the post-test. α wave power (parietal region): significant differences in post-test α wave power between the experimental and control groups (*p* < 0.001, Cohen's *d* = 1.372, medium to large effect), with the experimental group showing significantly higher power. α wave power (central region): significant differences in post-test α wave power between the experimental and control groups (*p* < 0.01, Cohen's *d* = 1.372, large effect), with the experimental group showing significantly higher power. α wave power (prefrontal region): Significant differences in post-test α wave power between the experimental and control groups (*p* < 0.001, Cohen's *d* = 1.835, large effect), with the experimental group showing significantly higher power. β wave power (parietal region): significant differences in post-test β wave power between the experimental and control groups (*p* < 0.01, Cohen's *d* = 0.955, large effect), with the experimental group showing significantly higher power. β wave power (central region): significant differences in post-test β wave power between the experimental and control groups (*p* < 0.001, Cohen's *d* = 1.266, large effect), with the experimental group showing significantly higher power. β wave power (prefrontal region): Significant differences in post-test β wave power between the experimental and control groups (*p* < 0.001, Cohen's *d* = 1.087, large effect), with the experimental group showing significantly higher power. θ wave power (parietal region): no significant difference in post-test θ wave power between the experimental and control groups (*p* > 0.05, Cohen's *d* = 0.527, small effect), but the experimental group showed significantly higher power. θ wave power (central region): no significant difference in post-test θ wave power was observed between the experimental and control groups (p > 0.05, Cohen's d = 0.539). θ wave power (prefrontal region): Significant differences in post-test θ wave power between the experimental and control groups (*p* < 0.05, Cohen's *d* = 0.804, large effect), with the experimental group showing significantly higher power. As shown in Table 2, mixed repeated measures ANOVA revealed significant main effects of time for α, β, and θ wave power, with the experimental group showing greater improvements across multiple brain regions. To visually present EEG changes before and after the intervention, Δ % topographic maps and radar plots for each channel's power were further generated (see Figures 5, 6).

**Table 2:** Changes in EEG power across brain regions before and after the intervention and the results of repeated measures ANOVA.

**Measure**	**Group**	**Pretest (M ±SD)**	**Posttest (M ±SD)**	Main Effect (partial ***η***^**2**^)		***F* [group] × [time] (partial η^2^)**
				**Group**	**Time**	
α Wave power (parietal region)	Experimental	0.02912 ± 0.00275	0.03343 ± 0.00334	7.905^**^ (0.294)	725.100^***^ (0.974)	133.634^***^ (0.876)
	Control	0.02820 ± 0.00173	0.02974 ± 0.00183			
α Wave power (central region)	Experimental	0.02979 ± 0.00188	0.03415 ± 0.00219	13.719^**^ (0.419)	399.898^***^ (0.955)	21.047^***^ (0.000)
	Control	0.02928 ± 0.00159	0.03063 ± 0.00161			
α Wave power (prefrontal region)	Experimental	0.02979 ± 0.00199	0.03304 ± 0.00214	0.491 (0.025)	1234.460^***^ (0.985)	267.493^***^ (0.934)
	Control	0.02883 ± 0.00180	0.03126 ± 0.00211			
β Wave power (parietal region)	Experimental	0.07883 ± 0.00543	0.08657 ± 0.00568	2.171 (0.103)	309.296^***^ (0.942)	141.428^***^ (0.882)
	Control	0.07950 ± 0.00423	0.08148 ± 0.00495			
β Wave power (central region)	Experimental	0.07980 ± 0.00394	0.08746 ± 0.00454	3.334 (0.149)	510.720^***^ (0.964)	327.687^***^ (0.945)
	Control	0.08040 ± 0.00459	0.08157 ± 0.00478			
β Wave power (prefrontal region)	Experimental	0.07928 ± 0.00473	0.08691 ± 0.00517	1.737 (0.084)	612.864^***^ (0.970)	201.029^***^ (0.914)
	Control	0.08046 ± 0.00426	0.08197 ± 0.00383			
θ Wave power (parietal region)	Experimental	0.02110 ± 0.00169	0.02001 ± 0.00157	0.183 (0.010)	49.502^***^ (0.723)	83.525^***^ (0.815)
	Control	0.02078 ± 0.00171	0.02087 ± 0.00169			
θ Wave power (central region)	Experimental	0.02127 ± 0.00124	0.02013 ± 0.00117	0.331 (0.017)	155.529^***^ (0.891)	144.009^***^ (0.883)
	Control	0.02095 ± 0.00140	0.02085 ± 0.00148			
θ Wave power (prefrontal region)	Experimental	0.02152 ± 0.00118	0.02037 ± 0.00114	1.604 (0.078)	149.134^***^ (0.887)	107.873^***^ (0.850)
	Control	0.02142 ± 0.00120	0.02139 ± 0.00139			

^*^p < 0.05, ^**^p < 0.01, ^***^p < 0.001.

## 4 Discussion and analysis

### 4.1 The effect of acute aerobic exercise on stroop and 2-back task performance

This study found that acute aerobic exercise significantly enhanced executive function in smartphone-addicted adolescents. The experimental group showed an increase in Stroop task accuracy from 71.3 to 78.4% (*p* < 0.001, *d* = −2.67) and a reduction in reaction time by approximately 140 ms (*p* < 0.001, *d* = 3.17), while the control group showed no significant changes. In the 2-back task, the experimental group's accuracy improved by around 9% (*p* < 0.001, *d* = −3.26), and reaction time decreased by more than 130 ms (*p* < 0.001, *d* = 3.29), with significant improvement over the control group in the post-test (*p* < 0.01, *d* = 0.93). In contrast, while the Stroop interference effect did not show significant differences in accuracy (*p* > 0.05), the reduction in reaction time was statistically significant (*p* < 0.05, *d* = 0.23). These results indicate that acute aerobic exercise primarily improves task performance by enhancing processing speed and the efficiency of cognitive resource allocation, with a relatively limited effect on the more complex process of conflict inhibition.These findings align with those of Chang et al. (15) and Kao et al. (16), who found that moderate-intensity acute exercise significantly improves reaction speed and accuracy, but has no notable effect on conflict resolution. Similarly, the study by Ren et al. (17) showed that exercise could improve inhibitory function in children with Attention Deficit Hyperactivity Disorder (ADHD), with the improvement primarily depending on better attention allocation and cognitive resource mobilization, rather than a direct enhancement of inhibition itself. This study replicates a similar pattern in adolescents with smartphone addiction.

This study further reveals the specificity of the exercise effect in the smartphone addiction population. Previous research has shown that individuals with smartphone addiction exhibit notable deficits in cognitive functions such as attention control and working memory (18, 19). The necessity of intervention is underscored by the stress-insomnia chain reaction triggered by addiction (20). Aerobic exercise serves as a feasible strategy, as physical activity can reduce addictive tendencies by bolstering self-esteem (21) and mitigating harmful impacts on social anxiety and sleep (22). The results of this study demonstrate that improvements in these individuals following exercise intervention even exceed the findings from previous studies in the general population. Specifically, in the 2-back task accuracy, the experimental group showed a large effect size and significantly outperformed the control group. This result aligns with Liu et al. (23), who found that individuals with smartphone addiction typically exhibit executive control deficits related to prefrontal cortex function. Exercise can compensate for these deficits by activating reward-related brain regions and enhancing prefrontal-dependent inhibitory control, which positively impacts addictive behavior improvement. In other words, exercise generally enhances performance in healthy individuals, but may show more significant restorative effects in addictive populations. This conclusion is consistent with the research of Kujach et al. (24), who suggested that exercise improves prefrontal cortex processing efficiency, and aligns with the findings of Zheng et al. (25), who found that acute moderate-intensity exercise significantly improves working memory performance. Therefore, individuals with smartphone addiction, due to their lower baseline cognitive functions, are more likely to experience significant functional improvement after exercise.

From a neurophysiological perspective, acute aerobic exercise enhances the excitability and blood flow of the dorsolateral prefrontal cortex (DLPFC) (26, 27) and modulates the activity of cognitive control-related brain regions such as the anterior cingulate cortex (ACC) (28). It also promotes the release of neurotransmitters such as dopamine and norepinephrine (29), improving the signal-to-noise ratio of the nervous system and the efficiency of attention resource allocation. This mechanism directly affects the maintenance and updating of working memory, which typically leads to more significant improvements in N-back tasks (such as 2-back) (30). In contrast, the Stroop interference effect mainly depends on the inhibitory control network composed of the ACC and inferior frontal gyrus (IFG), and short-term exercise may not produce significant changes in this more complex system (31). Therefore, the observed pattern in this study—overall accuracy and reaction time showing significant improvements, but limited enhancement in conflict-specific measures—not only aligns with existing research but also provides empirical evidence for explaining the differential effects of acute exercise on various cognitive processes.

### 4.2 EEG power changes induced by acute aerobic exercise and discussion of neurophysiological mechanisms

At the EEG power spectral level, this study found that acute aerobic exercise significantly and specifically modulated the neural oscillatory patterns of smartphone-addicted adolescents. Specifically, α and β wave power increased significantly in the prefrontal, central, and parietal regions, while θ wave power decreased in the prefrontal and central regions. This bidirectional modulation pattern, characterized by high-frequency upregulation and low-frequency downregulation, aligns with the neural oscillation framework linking exercise to cognitive enhancement (32) and provides empirical support for explaining the electrophysiological mechanisms underlying the immediate cognitive improvements observed in addicted populations.

Repeated measures ANOVA and independent sample *t*-test results showed that these changes not only demonstrated significant time effects but also exhibited robust differences between the experimental and control groups: the increase in α and β wave power reached large effect sizes (*d* = 0.95–1.80) in between-group comparisons, while the decrease in θ wave power was significantly different in the prefrontal region (*d* = 0.80). Topographic analysis of the power change percentage (Δ %) further revealed that the exercise-induced neural activity changes were not uniform across the entire brain, but were primarily concentrated in the prefrontal-central cortical regions. This spatial distribution closely aligns with key hubs of higher cognitive functions, including attention control and working memory, suggesting that acute aerobic exercise may restore and optimize the neural function of smartphone-addicted adolescents by selectively modulating the relevant neurocognitive networks.

#### 4.2.1 Enhancement of α wave power and inhibitory control mechanism

The significant increase in α wave power is a core feature of the EEG results in this study. After the intervention, the experimental group showed a significant upregulation of α wave power in the prefrontal, central, and parietal regions, with the most prominent change observed in the prefrontal region (*d* = 1.83, *p* < 0.001). Topographic analysis of the power change percentage (Δ %) revealed high-value “hot spots” in the prefrontal-central region. Within the framework of information gating and inhibitory control, this pattern can be interpreted as an enhancement of inhibitory gating to meet the demand of “focusing attention and inhibiting irrelevant inputs.” This increase in prefrontal-parietal α wave synchronization helps to reduce task-irrelevant noise and enhance the relative weight of useful signals (33, 34).

Compared to acute exercise studies in healthy individuals (35, 36), this study observed a more pronounced upregulation in the smartphone addiction population, with stable large effect sizes between groups (prefrontal/central/parietal regions, *p* < 0.01; *d* = 1.3–1.8). This result aligns with evidence that individuals with addiction have lower baseline α wave power and insufficient inhibitory control (37). Paired sample *t*-tests revealed a very large effect size in the experimental group (prefrontal α wave: *d* = 8.2), indicating significant individual-level changes, while between-group effects provided a conservative and robust comparison. These analyses complement each other, depicting the “individual improvement trajectory” and “intervention-control difference.”

Mechanistically, acute aerobic exercise enhances the metabolic conditions of the prefrontal-parietal cortex by increasing cerebral blood flow and oxygenation (38, 39). It also improves the signal-to-noise ratio and gating gain of prefrontal circuits by enhancing the phasic release of norepinephrine and dopamine (40, 41) and may strengthen α wave synchronization via the thalamocortical pathway (42). Additionally, μ-α waves in the central region (sensorimotor α) are closely related to the inhibitory mechanisms of the sensorimotor system. Haegens et al. (43) found that α oscillations have a rhythmic inhibitory effect on neuronal firing, with a decrease in α power (event-related desynchronization, ERD) associated with higher firing rates, supporting the inhibitory function of α waves in shielding irrelevant channels. Meanwhile, β waves are believed to help maintain the brain's stable preparatory state (44).

It should be noted that classical literature also reports α wave desynchronization (ERD) during task execution (45), where local α power decreases during the task to release neural channels. However, this phenomenon does not conflict with the results of this study: the present study focuses on the resting or transitional state following the intervention, where the system may generally increase α wave inhibition. During the task phase, local α waves can support information processing via desynchronization. If the “gating” interpretation holds, the coherence and directional connectivity of prefrontal-parietal α waves should significantly increase post-intervention and correlate positively with subsequent interference inhibition performance. This prediction aligns with previous findings on the coupling of α wave coherence and executive control (46).

#### 4.2.2 Enhancement of β wave power and cortical activation effects

Complementing the α wave's “inhibitory gating” mechanism, the significant increase in β wave power (13–30 Hz) represents another facet of “maintenance and preparation.” After the intervention, the experimental group showed the strongest upregulation of β wave power in the central and prefrontal regions (independent sample *d* = 1.27 and 1.09, respectively; *p*s < 0.001). Topographic analysis of the power change percentage (Δ %) revealed high-value “hot spots” along the central-to-frontal midline. Functionally, β rhythms are widely regarded as key oscillations that maintain the current status quo (i.e., sustaining existing motor or cognitive states) and play a core role in sensorimotor integration (47, 48). Their enhancement typically signifies a more stable preparatory or conserved state of the system, facilitating sustained attention and rapid response. Addictive and attention-deficit samples commonly exhibit low β waves and elevated θ/β ratios (49), correlating with attention instability and delayed responses. Therefore, the large effect of β wave upregulation in this study indicates a neural marker of “enhanced stability and preparation.”

From an evidence perspective, paired sample *t*-tests revealed a very large effect size (central region β wave: *d* = 5.7), indicating a steep individual improvement trajectory. Correspondingly, the robust between-group large effect further rules out the possibility that the results were merely due to statistical amplification. Mechanistically, exercise-induced activation of the locus coeruleus-norepinephrine (LC-NE) system and dopamine (DA) gain in the midbrain can promote phase coherence in sensorimotor circuits, especially in the β/γ frequency range (50, 51). Simultaneously, the pulmonary-driven increase in cerebral blood flow and enhanced cortical-cortical and cortical-thalamic coupling (39) provide the energy and structural conditions for brain rhythm synchronization. It is important to note that central β waves are also associated with GABA levels in the primary motor cortex (M1) and the cortical inhibition state (52); acute exercise has been shown to modulate the balance between GABA and glutamate (53), promoting a reset of the inhibitory-excitatory balance, which manifests as the enhancement of sensorimotor β waves.

From a dynamic perspective, β wave upregulation occurs alongside the increase in α wave gating tone and is accompanied by the downward shift in the θ frequency band, together forming a tripartite oscillatory pattern of “outward alertness—selective inhibition—maintenance of set.” It should be distinguished that, if the observation window occurs during the immediate phase of motor preparation/execution, β waves typically show event-related desynchronization (ERD), i.e., a decrease. However, the present study focused on the resting or transitional state following intervention, where the upregulation is more consistent with the interpretation of “baseline template improvement, ready for immediate use” (48). If the “set” framework holds, the coherence of central-prefrontal β waves post-intervention should increase, along with directional coupling between M1 and the prefrontal cortex, and it should be negatively correlated with subsequent reduced response variability and lower error rates (44, 54).

#### 4.2.3 Decrease in θ wave power and regulation of arousal levels

Relatively speaking, the directional decrease in θ wave power (4–8 Hz) in the prefrontal and central regions completes the third piece of the “inverse relationship” puzzle alongside the upregulation of α/β waves. The inter-group difference in prefrontal θ waves reached a medium to large effect size (*d* = 0.80, *p* < 0.05), and topographic analysis of the power change percentage (Δ %) revealed a stable “cold zone” along the frontal midline. The functional interpretation of θ waves requires distinguishing between different states: during task execution, frontal midline θ (fm-θ) waves are widely considered a neural marker of cognitive control and monitoring (55), while during rest or recovery, higher θ power is typically associated with endogenous synchronization, mind-wandering, and a low-arousal state (56). Given that this study's EEG recording included a resting window after the intervention, we interpret the decrease in θ waves as a reduction in slow-frequency spontaneous activity and an increase in outward arousal, consistent with the overall pattern of α/β wave upregulation.

More importantly, in the addiction phenotype, an elevated θ/β ratio (TBR) is typically seen as an electrophysiological marker of low arousal and a high tendency for distraction (57). This study also observed a decrease in θ waves and an increase in β waves, suggesting that acute exercise may have a bidirectional corrective effect on abnormal TBR. Mechanistically, exercise-induced activation of the locus coeruleus-norepinephrine (LC-NE) system and dopamine (DA) gain in the midbrain can suppress excessive slow-frequency activity, enhancing cortical gain and selectivity (40), while θ-γ coordination between the prefrontal cortex (PFC) and hippocampus during rest may shift from a “persistent activation” state to “demand-driven coordination,” thereby reducing ineffective synchronization and freeing up θ-γ coordination space for subsequent task periods (58). This pattern of results supports the understanding of θ wave changes as a transition from endogenous dissociation to outward mobilization, rather than a simple weakening of “task-required control.”

From an evidence perspective, paired sample *t*-tests showed a very large effect size for prefrontal θ waves (*d* = 4.6), indicating significant individual-level improvement trajectories. The medium to large between-group effects further suggest that this improvement is robust in the group comparison. If the “TBR correction/outward mobilization” hypothesis holds, then after the intervention, resting-state arousal indicators such as pupil diameter and electro dermal activity should increase, the fractional amplitude of low-frequency fluctuations (fALFF) in resting-state functional low-frequency amplitude should decrease in θ components, and the reduction in TBR should positively correlate with the enhancement of task-phase fm-θ phase coherence. These provide specific predictions for cross-modal validation (55).

#### 4.2.4 Integrated mechanism model and theoretical explanation

By incorporating the hierarchy of effect sizes into the oscillatory mechanism framework, we establish a unified and testable “bidirectional modulation–network gain” model. The extremely large effect sizes (*d* values) observed in paired sample tests for the experimental group suggest that individuals' trajectories from low baseline to resetting a more efficient oscillatory state are notably steep. The effect sizes (α/β wave *d* = 0.95–1.84; prefrontal θ wave *d* = 0.80) in independent samples provide a more conservative reference estimate, indicating robust external differences at the group level. These two aspects are complementary: the former emphasizes the “magnitude of improvement,“ while the latter depicts the ”intervention advantage.”

From a physiological mechanism perspective, acute aerobic exercise selectively upregulates mid-to-high frequency (α/β waves) and downregulates slow frequency (θ waves) in prefrontal-central hubs through immediate increases in cerebral blood flow and oxygenation (39), gain modulation of the catecholaminergic ascending system (50), and thalamocortical gating and “communication through coherence” mechanisms (51). This process reduces the θ/β ratio (TBR), increases the network signal-to-noise ratio and plasticity window, pushing the neural system toward a “higher alertness—better gating—more stable set” working point. This oscillatory reconfiguration pattern resonates with acute exercise evidence in healthy individuals (35, 59, 60) and shows high sensitivity and compensatory characteristics in low-baseline cognitive populations: when the baseline is low and network noise is high, exercise-induced gain modulation more easily leads to significant power reorganization and a better available state.

From a methodological and theoretical perspective, the following specific predictions can be made: after the intervention, the coherence of α/β waves between the prefrontal cortex (PFC), anterior cingulate cortex (ACC), and primary motor cortex (M1) should significantly increase; the θ/β ratio (TBR) should decrease; arousal indicators such as pupil diameter and electrodermal activity should increase; oxygenated hemoglobin concentration (HbO) in the prefrontal-central region should rise as measured by functional near-infrared spectroscopy (fNIRS). The coupling relationships between these cross-modal indicators and EEG power change percentage (Δ %) will serve as key empirical evidence to validate the “oscillation-network-chemical” triad mechanism chain (61).

Overall, the bidirectional modulation results of EEG power suggest that acute aerobic exercise does not merely increase cortical activation but achieves optimization of the cognitive control network through frequency band-specific dynamic balance. The upregulation of α and β waves reflects enhanced information gating and executive preparation, while the downregulation of θ waves indicates the restoration of alertness and correction of abnormal synchronization. This selective neural oscillatory modulation provides a new perspective for understanding the neurophysiological mechanisms through which exercise interventions improve addictive behaviors and lays a theoretical foundation for future exploration of the neural mechanisms underlying exercise prescriptions.

### 4.3 Practicable implications

The findings of this study offer important practical guidance for the intervention and prevention of smartphone addiction among adolescents. As a non-pharmacological and low-cost intervention, a single 20-min bout of moderate-intensity aerobic exercise can immediately alleviate cognitive deficits, suggesting that schools and families could implement brief “exercise breaks” to help adolescents with addiction tendencies quickly regain cognitive focus and improve learning efficiency. Furthermore, the identified oscillatory pattern of high-frequency upregulation (increased α and β waves) and low-frequency downregulation (decreased θ waves) serves as a potential neurobiological marker for evaluating the effectiveness of exercise prescriptions. Future programs could utilize portable EEG devices to monitor these brain rhythm changes, enabling the development of personalized exercise programs tailored to the specific neurophysiological needs of addicted adolescents.

### 4.4 Limitations and future research

This study revealed the improvement of cognitive performance and EEG activity in smartphone-addicted adolescents through acute aerobic exercise, but there are several limitations. The study only involved male adolescents, so the results may not be broadly applicable to other populations. The experiment only assessed the short-term effects of acute exercise and lacked follow-up data on long-term interventions. While the experimental tasks effectively measured cognitive function, they did not comprehensively cover all related cognitive processes. EEG data analysis focused solely on the prefrontal, central, and parietal regions, without exploring the impact of other brain regions. Future research using high-density EEG or multi-modal imaging could provide a more comprehensive mapping of whole-brain network reconfiguration.

## 5 Conclusion

This study demonstrates that acute aerobic exercise effectively enhances executive function and optimizes neural activity in adolescents with smartphone addiction. Behavioral results indicate significant improvements in inhibitory control and working memory, characterized by increased processing speed and accuracy. Electro physiologically, exercise induced a bidirectional oscillatory reconfiguration—upregulating mid-to-high frequency (α/β) power while downregulating slow frequency (θ) power in prefrontal-parietal hubs. These findings suggest that aerobic exercise facilitates the restoration of cognitive control networks by enhancing neural resource mobilization. Overall, this research provides robust empirical evidence for the “oscillation-network” mechanism and supports the implementation of exercise-based interventions as a viable, non-pharmacological strategy to ameliorate cognitive deficits and addictive behaviors in the adolescent population.

## Data Availability

The raw data supporting the conclusions of this article will be made available by the authors, without undue reservation.
